# Osteoarthritic Subchondral Bone Release Exosomes That Promote Cartilage Degeneration

**DOI:** 10.3390/cells10020251

**Published:** 2021-01-28

**Authors:** Xiaoxin Wu, Ross Crawford, Yin Xiao, Xinzhan Mao, Indira Prasadam

**Affiliations:** 1 Department of Orthopaedic Surgery, the Second Xiangya Hospital, Central South University, Changsha 410011, China; x42.wu@hdr.qut.edu.au; 2 Institute of Health and Biomedical Innovation, School of Mechanical, Medical and Process Engineering, Queensland University of Technology, Brisbane 4059, Australia; r.crawford@qut.edu.au (R.C.); yin.xiao@qut.edu.au (Y.X.); 3 Orthopedic Department, the Prince Charles Hospital, Brisbane 4059, Australia; 4 Australia-China Centre for Tissue Engineering and Regenerative Medicine, Queensland University of Technology, Brisbane 4059, Australia

**Keywords:** exosomes, subchondral bone, cartilage, osteoarthritis, miRNA, cell-interactions

## Abstract

Altered subchondral bone and articular cartilage interactions have been implicated in the pathogenesis of osteoarthritis (OA); however, the mechanisms remain unknown. Exosomes are membrane-derived vesicles that have recently been recognized as important mediators of intercellular communication. Herein, we investigated if OA subchondral bone derived exosomes alter transcriptional and bioenergetic signatures of chondrocytes. Exosomes were isolated and purified from osteoblasts of nonsclerotic or sclerotic zones of human OA subchondral bone and their role on the articular cartilage chondrocytes was evaluated by measuring the extent of extracellular matrix production, cellular bioenergetics, and the expression of chondrocyte activity associated marker genes. Exosomal microRNAs were analyzed using RNA sequencing and validated by quantitative real-time PCR and loss-of-function. In coculture studies, chondrocytes internalized OA sclerotic subchondral bone osteoblast derived exosomes and triggered catabolic gene expression and reduced chondrocyte-specific marker expression a phenomenon that is often observed in OA cartilage. RNA sequencing and miRNA profiling have identified miR-210-5p, which is highly enriched in OA sclerotic subchondral bone osteoblast exosomes, triggered the catabolic gene expression in articular cartilage chondrocytes. Importantly, we demonstrate that miR-210-5p suppresses the oxygen consumption rate of chondrocytes, altering their bioenergetic state that is often observed in OA conditions. These effects were markedly inhibited by the addition of a miR-210-5p inhibitor. Our study indicates that exosomes released by OA sclerotic subchondral bone osteoblasts plays a critical role in progression of cartilage degeneration and might be a potential target for therapeutic intervention in OA.

## 1. Introduction

Crosstalk between articular cartilage and subchondral bone has been suggested to play a crucial role in the pathogenesis of osteoarthritis (OA) [1,2]. Indeed, a series of our previous studies have provided a strong evidence that the interaction of cartilage and bone cells in OA joints leads to the bidirectional cellular phenotypical changes, resulting in cartilage degeneration and subchondral bone sclerotic changes during OA progression [3,4,5,6]. However, the precise molecular mechanisms governing the altered cell–cell communication in OA have not yet been clarified.

Exosomes are bioactive, bilipid membrane nanovesicles of endosomal origin and are known to mediate cell-to-cell communication [7,8,9,10,11]. The inward budding of the plasma membrane produces multivesicular bodies (MVBs) which contain intraluminal vesicles (ILVs) [8,11,12]. The fusion of MVBs with the plasma membrane results in the release of ILVs into the extracellular environment as exosomes [8,11,13]. MVBs range from 50–1000 nm in diameter while exosomes range from 30–150 nm in diameter and are formed within MVBs [14]. As carriers of a variety of proteins, mitochondrial RNA (mtRNA), microRNAs (miRNAs), and noncoding RNAs (ncRNAs), exosomes play a crucial role in intercellular communication under both normal and pathological conditions [8,9,11,15]. Several previous studies have shown that exosomes can mediate cell-to-cell communication in both physiological and pathological conditions [16,17,18,19,20,21]. Furthermore, exosomes have the ability to carry messenger RNA (mRNA), miRNA, DNA, proteins, and lipids which can be transferred to recipient cells and alter their physiological functions [8,9,11,16,20,21]. In our previous studies we showed that the pathological effects of OA subchondral bone osteoblasts (SBOs) on articular cartilage chondrocytes (ACCs) is mediated via the mitogen-activated protein kinase (MAPK) signaling pathway [3]. However, the components secreted from OA SBOs that are responsible for modulating this biological function of ACCs, are still unknown.

In the present study we hypothesize that the altered crosstalk between OA cartilage and the subchondral bone microenvironment is mediated by exosomes. To verify this hypothesis, we designed this study to determine whether exosome uptake and transport can also occur between subchondral bone osteoblasts and articular chondrocytes, thereby assessing the application of exosomes as targeted drug carriers for the treatment of OA.

## 2. Materials and Methods

### 2.1. Human Tissue Collection and Cell Culture

**Figure 1:**
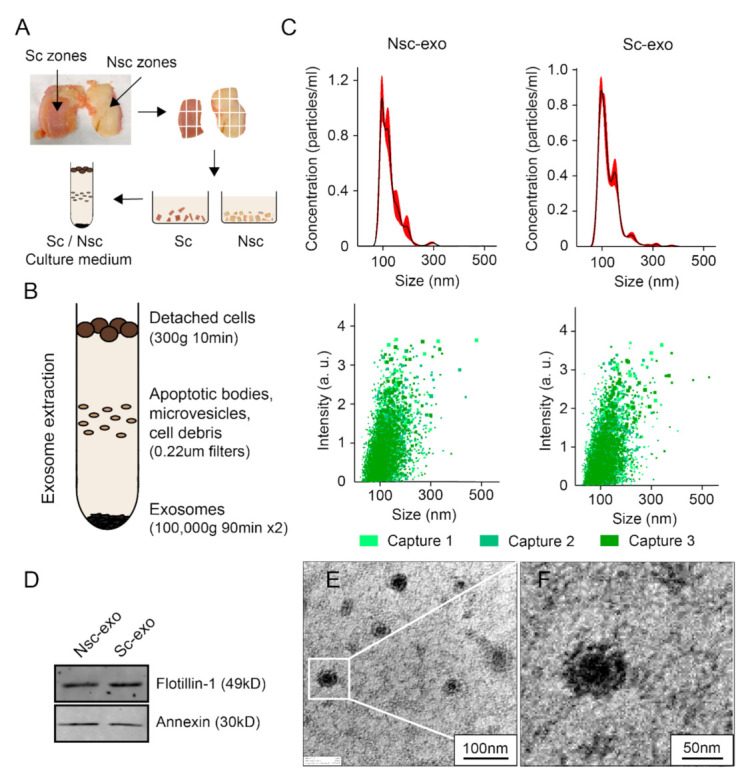
Methods and characterization of exosomes extracted from Nsc-SBOs and Sc-SBOs. (**A**,**B**) Exosomes were isolated from culture medium of Nsc-SBOs and Sc-SBOs. (**C**) Exosome size and concentration was measured using NS300 NanoSight (n = 3 patients). (**D**) Western blotting analysis of exosomal markers in Nsc-SBOs and Sc-SBOs exosomes (images representative of n = 3 patients). (**E**,**F**) TEM images of purified exosomes derived from SBOs. The images are representative of exosomes isolated from three different patients. Nsc, nonsclerotic; Sc, sclerotic; SBOs, subchondral bone osteoblasts; Nsc-exo, nonsclerotic SBO derived exosomes; Sc-exo, sclerotic SBO derived exosomes.

Human ethical approval for this project was granted by the Queensland University of Technology (QUT) and the St Vincent Private Hospital Ethics Committees (No. EC00324), and informed consent was obtained from all participating subjects. Subchondral bone was collected from the tibia surface from the patients who underwent primary total knee replacement surgery. The mean age of the patients 81 years and BMI range of 23.6–28.4 with no known other disease conditions (four females, two males). Osteoblasts were derived from nonsclerotic and sclerotic regions zones within the same joint. Nonsclerotic and sclerotic zones were characterized by a visual difference in their thickness and hardness and the osteoblast activity assessment as described by our team in previously published articles [4,5]. Nonsclerotic regions were covered by cartilage without surface irregularities, whereas sclerotic regions were denuded or covered by severely degraded cartilage (Figure 1A). After removing the overlying cartilage, subchondral bone was cut into small pieces and washed 3–4 times with phosphate-buffered saline (PBS) to remove the blood debris. SBOs were cultured and characterized as described previously in low glucose Dulbecco’s Modified Eagle’s Medium (DMEM) with 10% fetal bovine serum (FBS) and 1% Penicillin-streptomycin (PS) (complete medium) [4,5]. Chondrocytes were isolated from visually undamaged areas using type II collagenase enzymatic digestion method as described previously [5,6].

### 2.2. Preparation of Conditioned Medium and Isolation of Exosomes

Passage 1 SBOs were grown until 80% confluence and the medium was replaced by serum free DMEM. Conditioned medium (60 mL in total) was collected after 24 h of incubation and filtered through a 0.22 μm filter sterilizer. The collected medium was defined as nonsclerotic SBOs or sclerotic SBOs cultured conditioned medium (CM) and was stored at −80 °C before use in the subsequent experiments (Figure 1A). In this study, we used ultracentrifugation method to isolate exosomes. The SBOs CM was centrifuged using a Beckman Coulter Microfuge 18 Centrifuge (Bechman Coulter Life Sciences, Indianapolis, United States) at 300× *g* at 4 °C for 10 min to remove detached cells and was filtered through 0.22 μm filters (Sarstedt, Numbrecht, Germany) to remove contaminating apoptotic bodies, microvesicles, and cell debris (Figure 1B). Clarified CM was then centrifuged in a Beckman Coulter Optima^TM^ L-90K Ultracentrifuge at 100,000× *g* at 4 °C for 90 min with a Type SW41 rotor to pellet exosomes. The exosome-containing pellets were resuspended in ice-cold PBS. A second round of ultracentrifuge using Beckman Coulter OptimaTM MAX-80XP Ultracentrifuge at 100,000× *g* at 4 °C for 90 min with a Type TLA 110 rotor was carried out, and the final exosome pellets were resuspended in PBS after carefully removing the supernatant [22] (Figure 1B).

### 2.3. Size Distribution Analysis

The size distribution and particle concentration of exosomes were determined using nanoparticle tracking analysis. NS300 NanoSight (ATA Scientific, Sydney, Australia) fitted with a NS300 flow-cell top plate and a 405 nm laser was used. The 1mL syringes (BD, Franklin Lakes, NJ, USA) were used as sample pump. Exosomes were diluted and analyzed at camera levels of 13 at 25 °C. Automatic settings for the maximum jump distance and blur settings were utilized. The detection threshold for all samples was 2. The NanoSight sample pump was set to 50. Exosomes (1:10) were loaded into the sample chamber prior to video recordings. Data was analyzed on the NTA software 3.0 (ATA Scientific).

### 2.4. Transmission Electron Microscopy (TEM)

Purified exosome morphologies were observed using a transmission electron microscope. Exosomes were suspended and loaded onto a carbon/formvar-coated copper TEM grids (Emgrid Australia, Adelaide, Australia) with a 7 µL drop. Once dry, the grids were stained with 1% uranyl acetate for 30 s–1 min depending on the concentration of exosomes and then left to dry before images were obtained under transmission electron microscopy (JEM-1400, JOEL, Tokyo, Japan). The diameter of the exosome was evaluated by the ImageJ software.

### 2.5. Western Blotting

Western blots for common exosomal markers were performed as previously described [22]. Briefly, exosome isolations were lysed in reducing sample buffer (0.25 M Tris-HCl (pH 6.8), 40% glycerol, 8% sodium dodecyl sulfate (SDS), 5% 2-mercaptoethanol and 0.04% bromophenol blue) or nonreducing sample buffer (without 2-mercaptoethanol) and boiled for 10 min at 95 °C. Proteins were resolved by SDS-PAGE (SDS-polyacrylamide gel electrophoresis), transferred to polyvinylidene fluoride membranes, blocked in 5% nonfat powdered milk in PBS-T (0.5% Tween-20) and probed with antibodies using Exosomal Marker Antibody Sampler Kit (Cell Signaling, Lot #74220). Protein bands were detected using Odyssey infrared Imaging System (LI-COR Biotechnology, USA).

### 2.6. MicroRNA-Sequencing

Total RNA from the purified exosomes of nonsclerotic SBOs or sclerotic SBOs (n = 3) was extracted using TRIzol Reagent (Molecular Research Centre, TR118-500) following the manufacturer’s instructions. Small RNA cDNA libraries were generated using M-MLV Reverse Transcriptase (Promega, M1705) and applied to the Illumina HiSeq2500 (Illumina, San Diego, CA, USA) 50nt single-end sequencing. Within each sample, reads from miRNA genes were normalized and scaled to counts per million. Statistical analysis and clustering were performed using the DESeq2 algorithm and pheatmap in R software (|log2Ratio| ≥ 2, FDR ≤ 0.001 were significant). Gene Ontology analysis and Kyoto Encyclopedia of Genes and Genomes Pathway analysis were performed to predict microRNA target genes.

### 2.7. RNA Extraction and Quantitative Real-Time PCR

Total RNA was extracted using TRIzol reagent (Thermo Fisher, 15596018). RNA quantity and quality were assessed in a NanoDrop-100 spectrophotometer (Termo Scientifc, Scoresby, VIC, Australia). cDNA synthesis from 500 to 1000 ng of total RNA and real-time PCR were performed using miScript PCR Starter Kit (Qiagen, Lot #218193) according to manufacturer’s protocol. Quantitative measurements of all primers used in this study were determined using (2−ΔΔCt) method, and miR-92a-3p expression was used as the internal control using the QuantStudio Real-Time PCR system (Applied Biosystems, Thermo Scientific, Scoresby, VIC, Australia) [23,24]. The primer for miRNA were miScript Primer Assays (Qiagen) and the sequence are shown in Supplementary Materials Table S1.

### 2.8. Exosome Labeling with PKH67

Purified exosomes were prelabeled with PKH67 Green Fluorescent Cell Linker Mini Kit (Sigma-Aldrich, Sydney, Australia). Briefly, 25 µL of exosome solution was mixed with 225 µL Diluent C (Part A) and 1 µL PKH67 ethanolic dye solution was mixed with 250 µL Diluent C (Part B). Part A was quickly added to Part B and gently mixed before incubation at room temperature for 1–5 min. Staining was interrupted by adding 1 mL DMEM medium with 1% no-exosome FBS. After that, the labeled exosomes were incubated with chondrocytes for 24 h at 37 °C. Samples were fixed with 4% paraformaldehyde (Sigma-Aldrich) and mounted on glass slides with ProLong^®^ antifade reagents (Life Technologies, Thermo Fisher, Melbourne, Australia). Images were captured using a confocal laser scanning microscope (Nikon A1R Confocal, Amsterdam, The Netherlands).

### 2.9. Cell Proliferation Assay

Cell proliferation was determined using CyQUANT proliferation assay (Life Technologies, Thermo Fisher) and followed the manufacturer’s instruction. Then, 10, 20, 50 µg/mL of exosomes were added separately into the culture medium of chondrocytes. Fluorescence was measured after 48h.

### 2.10. Treatment of Human Chondrocytes with SBOs-Derived Exosomes

**Figure 2:**
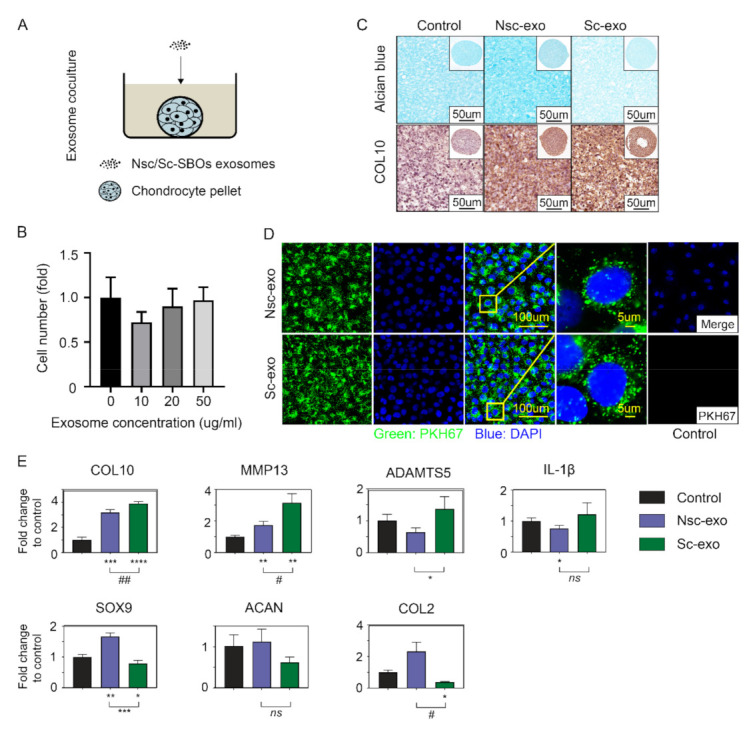
Sc-SBOs secreted exosomes contribute to cartilage degeneration. (**A**) Coculturing model of ACCs pellets and Nsc/Sc-SBOs exosomes. (**B**) Proliferation assay of ACCs cocultured with SBOs exosomes in 0/10/20/50 µg/mL (n = 3). (**C**) Alcian blue staining (top) and immunostaining (bottom) analysis of ACCs pellets cocultured with Nsc/Sc-SBOs exosomes (n = 3 patients). (**D**) PKH67-marked exosomes staining with ACCs (images are representative of n = 3 patients) and negative control. (**E**) Real-time PCR analysis of ACCs pellets cocultured with Nsc/Sc-SBO derived exosomes (n = 3), represented as the mean ± SD; *, *p* < 0.05, **, *p* < 0.01, ***, *p* < 0.001, ****, *p* < 0.0001; ##: Very significant, #: Significant; ns: Not significant

Macroscopically healthy cartilage was collected and washed with PBS for three times. After that, cartilage tissues were digested in 1 g/L of Type II Collagenase (Thermo Fisher) in DMEM to isolate ACCs for 6–8 h at 37 °C. After centrifugation (1200 rpm, 10 min), the supernatant was removed, and ACCs were cultured with complete culture medium at 37 °C with 5% CO_2_. Cells were grown to confluence before they were used for 3-dimensional pellet cultures in accordance with our published protocols (Figure 2A) [6]. A total of 20 µg/mL of exosomes were cocultured with 3-dimensional pellet and the chondrogenic culture medium (DMEM, High (4.5 g/L) Glucose (DMEM-HG, Invitrogen) supplemented with 1% Inulin-transferrin-selenium (Sigma), 1.25 mg/mL Bovine serum albumin (Sigma), 0.1 µM dexamethasone (Sigma), 0.1 mM ascorbic acid (Sigma), 1% PS (Gibco, Thermo Fisher), 10mM HEPES (Sigma), 0.1 mM L-proline (Sigma), 0.1 mM MEM Nonessential Amino Acids (Gibco, Thermo Fisher) and 10 ng/mL transforming growth factor-beta 1 (TGF-β1, Gibco, Thermo Fisher)) was changed twice a week. After coculturing for 14days, total RNA was extracted for gene expression test according to the protocol we published and some group of pellets from each group were embedded by paraffin for further staining [6].

### 2.11. Alcian Blue Staining

The 3-dimensional chondrocyte pellets paraffin sections were stained with 1% Alcian blue solution (Sigma-Aldrich) in accordance with our published protocols [25]. The images of whole pellets were photographed by SMZ745T, Nikon, and the slides were scanned by Pannoramic Scanner, 3DHISTECH. Staining intensity of the images was visually analyzed.

### 2.12. Immunohistochemistry Staining

Immunohistochemistry was performed according to our previously published study [26]. The 3-dimensional chondrocyte pellet paraffin sections were stained by anti-type X collagen (ab49945, Abcam, Cambridge, UK, dilution 1:800) antibodies to determine expression. The sections were incubated with corresponding fluorescent secondary antibody (ab150113, Abcam, Cambridge, UK, dilution 1:5000). Images were visualized using a Leica SP5 confocal microscope (Leica Microsystems, Sydney, Australia).

### 2.13. miRNA Transfection

ACCs were transfected according to the manufacturer’s instructions with Lipofectamine 2000 Reagent (Thermo Fisher). Briefly, Lipofectamine Reagent was mixed with Opti-MEM Medium (Gibco, Thermo Fisher) (Part A). A total of 14µg of miR-210-5p Mimics, Mimics Negative Control (Sigma-Aldrich), Inhibitor and Inhibitor Negative Control (Integrated DNA Technologies, Coralville, United States) were mixed with Opti-MEM Medium separately (Part B). Parts A and B were mixed with a 1:1 ratio and incubated for 5 min at room temperature (Part C). Following this, cell culture medium was changed to Part C medium and incubated for 6 h at 37 °C with 5% CO_2_. Following this, the cell culture medium was changed to normal culture medium. After 24 h, total RNA was extracted as described above and gene expression was detected in accordance with our previous publications [5,6,26,27].

### 2.14. Seahorse Analysis

The Seahorse Analyzer XF96 (Agilent, Santa Clara, CA, USA) was used to monitor oxygen consumption rate (OCR) and extracellular acidification rate (ECAR). Briefly, C28/I2 human chondrocytes cell line was seeded 10,000 cells per well and transfected with miR-210-5p Mimic, Inhibitor, and Negative controls separately for 24 h. One day before the test, 200 µL of XF Calibrator solution (Agilent) was added to each well of the XF sensor cartridge and incubated at 37 °C. Simultaneously, the Seahorse analyzer machine was turned on overnight. One hour before test, cell culture medium was changed to 200 µL per well of XF Base Medium (Agilent) with 1 mM Pyruvate, 2 mM Glutamine and 10 mM Glucose, pH = 7.4 for at least 45 min at 37 °C in humidified atmosphere without CO_2_. For Cell Mito Stress and Energy Phenotype analysis, 1 µM Oligomycin, 2 µM Carbonyl cyanide 4-(trifluoromethoxy)phenylhydrazone (FCCP), and 0.5 µM Rotenone/antimycin A were added to each injection port of the XF cartridge. After 15 min equilibration time, OCR and ECAR were accessed every 6 min (3 min mixing and 3 min measure), thrice after the addition of each compound.

### 2.15. Statistical Analysis

Statistical difference of the results was tested using unpaired t-test (for two-group comparisons) or ANOVA method (for multigroup comparisons) followed by a post hoc test [26]. All analyses were performed using GraphPad Prism 8 (San Diego, CA, USA) and *p*-values < 0.05 were significant. All data is presented as mean ± SD.

## 3. Results

### 3.1. Characterization of Exosomes Extracted from Nsc and Sc Zones Subchondral Bone Osteoblasts

Exosomes that were extracted from nonsclerotic SBOs and sclerotic SBOs using ultracentrifugation (100,000× *g* for 90 min at 4 °C) showed no significant differences in size, concentration, distribution, and markers. The mean size of SBOs derived exosomes is 115.5 nm in nonsclerotic zones, while 115.4 nm in sclerotic zones. The concentration of SBOs exosomes is 6.27 × 10^8^ particles/mL in nonsclerotic SBOs and 5.58 × 10^8^ particles/mL in sclerotic SBOs exosomes from 60 mL condition medium. Analyses of particle distribution between the both groups of exosomes (nonsclerotic SBOs vs. sclerotic SBOs) showed no difference (Figure 1C). Western blotting analyses of nonsclerotic SBOs and sclerotic SBOs exosomes indicated that they both expressed exosome specific markers Flotillin-1 and Annexin (Figure 1D). TEM images showed SBOs-derived exosomes as cap-shaped particles with double membranes (Figure 1E,F). Together these results indicate there is no significant phenotype difference between exosomes from nonsclerotic and sclerotic SBOs.

### 3.2. Exosomes Derived from Sclerotic SBOs Altered Chondrocyte Phenotype

Next, we determined the influence of nonsclerotic SBOs and sclerotic SBOs secreted exosomes on ACCs phenotype by performing proliferation analysis, Alcian blue staining for proteoglycans, immunostaining, and real-time PCR to quantitate the chondrocyte specific gene expression. Cell proliferation showed 20 µg/mL of exosomes is the minimum concentration while not affecting cell growth in the coculture model (Figure 2A,B). Coculture of ACCs with sclerotic SBOs exosomes showed decreased glycosaminoglycan (GAG) levels evidenced by Alcian blue and increased COL10 staining compared to nonsclerotic SBOs exosome culture group (Figure 2C). PKH67 staining showed ACCs (blue) phagocytosed both nonsclerotic SBOs and sclerotic SBOs exosomes (green) with no significant differences in uptake (Figure 2D). Hypertrophic markers of chondrocytes such as COL10, MMP13, and ADAMTS5 were significantly upregulated and chondrogenic markers such as SOX9 and COL2 were significantly downregulated (*p* < 0.05) in ACCs pellets cocultured with sclerotic SBOs derived exosomes (Figure 2E). All together results indicate exosomes derived from sclerotic SBOs suppressed the synthesis of chondrocytes extracellular matrix (ECM) and reduced chondrocyte-specific marker expression a phenomenon that is often observed in OA cartilage.

### 3.3. Exosomal miR-210-5p was Involved in the Abnormal Communication between Sclerotic SBOs and ACCs

**Figure 3:**
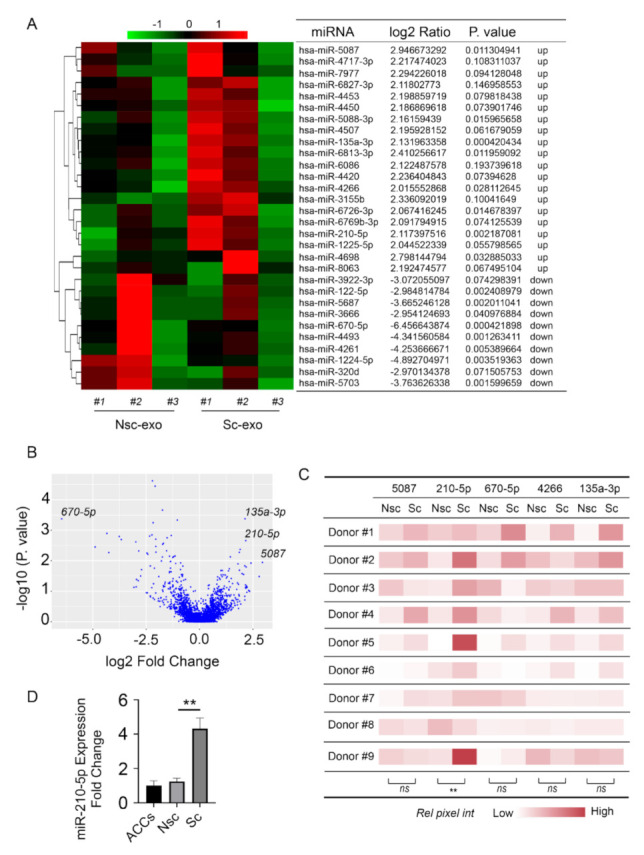
Exosomal miR-210-5p was involved in the abnormal communication between Sc-SBOs and ACCs. (**A**,**B**) MicroRNA sequencing analysis of Nsc/Sc-SBOs exosomes (n = 3 patients). (**C**) MiR-5087, miR-210-5p, miR-670-5p, miR-4266, and miR-135a-3p in Nsc/Sc-SBOs exosomes were measured using real-time PCR (n = 9). ** *p* < 0.01. (**D**) Real-time PCR analysis of ACCs treated with/without Nsc/Sc-SBOs exosomes (n = 3), represented as the mean ± SD; ** *p* < 0.01; ns: Not significant

To investigate the internal difference between nonsclerotic SBOs and sclerotic SBOs derived exosomes, exosomal microRNA expression was analyzed by RNA sequencing. Results indicate that the expression of several microRNAs was significantly altered in sclerotic SBOs derived exosomes compared to nonsclerotic SBOs (Figure 3A). From these altered miRNAs, the five most significantly altered microRNAs were chosen for further investigation based on *p*-values (Figure 3A,B). The expression of these five microRNAs was further confirmed using real-time PCR with nine different patient samples, which showed that miR-210-5p was consistently upregulated in sclerotic SBOs derived exosomes compared to nonsclerotic SBOs (Figure 3C). MicroRNA targeted pathway analysis showed that these microRNAs corresponded to cell cycle, PI3K-Akt, Hippo, and AGE-RAGE signaling pathway (Figure S1). The expression of miR-210-5p on ACCs coculture with/without nonsclerotic/sclerotic SBOs derived exosomes showed that sclerotic SBOs exosomes upregulated miR-210-5p in ACCs (Figure 3D). Together these results indicated that miR-210-5p was enriched in sclerotic SBOs derived exosomes, which might relate to cell cycle, PI3K-Akt, Hippo, and AGE-RAGE signaling pathway and contribute to the upregulation of miR-210-5p in ACCs cultured with sclerotic SBOs exosomes.

### 3.4. Upregulation of miR-210-5p Leads to Increased Chondrocytes Hypertrophic and Degradative Gene Expression and Cellular Aerobic Respiration Alteration

**Figure 4:**
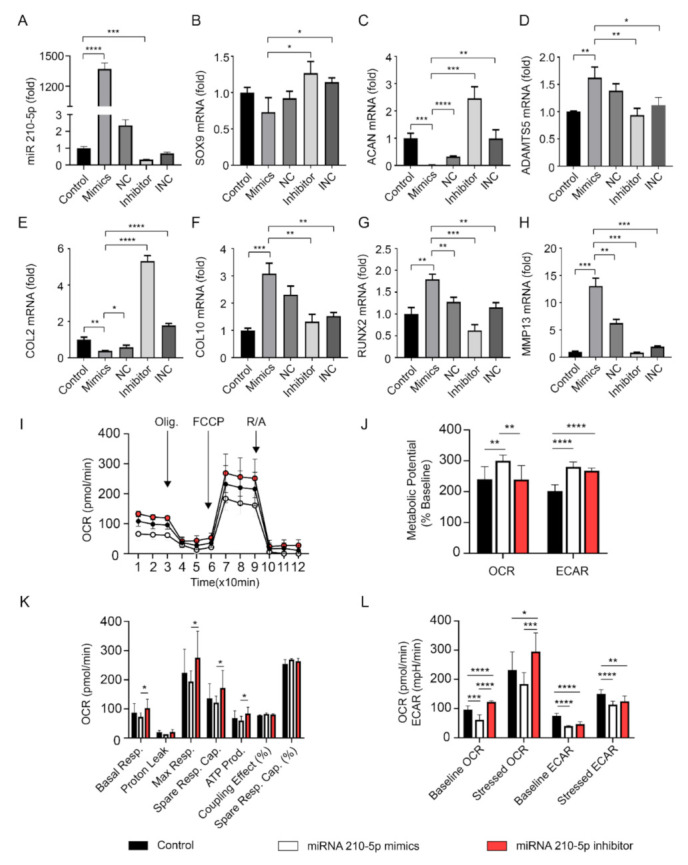
MiR-210-5p leads to hypertrophic change and cellular aerobic respiration alteration in chondrocytes. (**A**) The expression of miR-210-5p in chondrocytes after transfection (n = 3 patients). NC, negative control; INC, inhibitor negative control. (**B**–**H**) Real-time PCR of gene expression in chondrocytes after transfection (n = 3). (**I**–**L**) Seahorse mitochondrial stress and phenotype analysis of ACCs transfected by miR-210-5p mimics and inhibitor (n = 3). * *p* < 0.05, ** *p* < 0.01, *** *p* < 0.001, **** *p* < 0.0001.

Real-time PCR and Seahorse analysis were used to evaluate the effect of miR-210-5p on chondrocytes. The expression of miR-210-5p was significantly upregulated in the mimics group and downregulated in the inhibitor group (Figure 4A). Real-time PCR showed that chondrogenic markers such as SOX9, ACAN, and COL2 were downregulated and hypertrophic markers such as ADAMTS5, COL10, RUNX2, and MMP13 were upregulated in chondrocytes transfected with miR-210-5p mimics which were opposite in the inhibitor group (Figure 4B–H). In cellular energy metabolism analyses, miR-210-5p downregulated ATP production, basal, maximum, and spare respiration while upregulating metabolic potential in chondrocytes (Figure 4I–K). In energy phenotype analyses, miR-210-5p decreased the level of OCR in both baseline and stressed states but showed no significant difference in ECAR level (Figure 4L). Together these results showed that miR-210-5p might trigger the effect of sclerotic SBOs derived exosomes to ACCs regarding hypertrophic and degradative gene expression and cellular aerobic respiration alteration.

## 4. Discussion

Our previous studies have shown that SBOs can affect the behavior of the overlying cartilage under OA conditions [4,5]. This is the first study to identify the role of exosomes derived from nonsclerotic/sclerotic zones of subchondral bone on human articular chondrocytes. The three key findings from this study indicate that (1) OA sclerotic SBOs derived exosomes modulate the phenotype of chondrocytes by decreasing the extra cellular matrix production and increasing catabolic gene expression typically seen in OA cartilage. (2) miRNA sequencing analysis has identified exo-miR-210-5p, as highly enriched microRNA in OA sclerotic SBOs exosomes. (3) In chondrocytes, OA sclerotic SBOs cell-secreted miR-210-5p altered the energy metabolism and significantly affected the catabolic gene expression of chondrocytes.

Firstly, we successfully isolated the exosomes from nonsclerotic SBOs and sclerotic SBOs. The mean diameters of the exosomes from the two groups were around 115 nm, which is like those previously reported in literature [28]. These exosomes showed expression of Flotillin-1 and Annexin, which are standard markers that have been used to identify exosomes [29,30]. TEM images demonstrated that most of these vesicles we isolated were double membrane nanovesicles with diameters ranging between 50 and 150 nm. It is well known that subchondral bone osteoblasts can trigger catabolic effects in chondrocytes during OA progression. In coculture experiments we have seen that the presence of OA sclerotic SBOs significantly affected gene expression of anabolic and catabolic markers of chondrocyte cells. It is of interest noting the same effect can be observed if, instead of coculturing the cells, we treat the chondrocyte cells with the exosomes generated by the sclerotic SBOs. These results may indicate that exosomes mediate the altered cross talks during OA. By contrast, exosomes released by nonsclerotic SBOs seem to play a limited role in chondrocyte phenotypic changes suggesting that the altered exosome production during the cross-talk plays a central role in the progression of OA by induction of mechanisms that promote the catabolic effects. Several previous reports have shown the importance of exosomes, micro vesicles, and apoptotic bodies in synovial fluid and cartilage ECM are highly related to the regulation of joint homeostasis. From this study, it is evident that exosomes play a determining role in signaling between the cells of the joint micro-environment.

By carrying different kinds of protein, mRNA, and miRNA, exosomes were found to engage in cell-to-cell communication. Recent studies demonstrated that exosomal miRNAs serve as important signaling factors to regulate joint homeostasis. To identify the miRNA profile of exosomes derived from SBOs, high-throughput sequencing was used in the present study. A total of 2590 exosomal miRNAs were dysregulated in sclerotic SBOs compared with those in the nonsclerotic SBOs. One of these exosomal miRNAs, miR-210-5p was significantly upregulated. Different reports indicate that miR-210-5p is required for myelopoiesis, cognitive impairment and epithelial–mesenchymal transition [31,32,33]. For instance, miR-210-5p inhibits primitive myelopoiesis by silencing gata4/5/6 transcription factor genes [33]. MiR-210-5p also contributes to cognitive impairment through suppressing synaptosomal-associated protein of 25 KDa [32]. Sharing the same family with miR-210-5p, miR-210 has been widely reported involved in the regulation of chondrocytes and osteoblasts [34,35,36,37,38]. For example, miR-210 increased viability and suppressed apoptosis in hypoxic chondrocytes [35]. Overexpression of miR-210 promotes chondrocyte proliferation and extracellular matrix deposition in OA [34]. Zhang demonstrated that miR-210 suppressed inflammation in OA rat joints by inhibiting NF-κB signaling pathway [36]. MiR-210 also promotes osteoblastic differentiation while on the other hand, miR-210-3p inhibits osteogenic differentiation [37,38]. Besides, miR-210 was described as a potential marker for early prediction of OA, influencing vascular endothelial growth factor (VEGF) expression and majorly in osteonecrosis regions [39,40].

The underlying mechanisms for OA SBO exosome transfer of miR-210-5p on ACCs during OA remain to be fully elucidated. It has been reported by Liu that miR-210-5p could inhibit PIK3R5, which was a key regulator in the PI3K/AKT/mTOR signaling pathway [31]. The PI3K/AKT/mTOR signaling pathway is essential for normal metabolism of articular joints, the inhibition of which could induce autophagy and apoptosis in ACCs that lead to OA [41,42]. Ren et al. also demonstrated that miR-210-5p could target Snap25 using a rat model [32]. Snap25 was found to be an important gene involved in metabolic and neural diseases such as obesity, diabetes, and Alzheimer’s disease [43,44]. It was reported that the reduced expression of Snap25 caused by miR-210-5p could also suppress the PI3K/AKT signaling pathway which might be one of the possible mechanisms contributing to OA progress [43].

In addition, our findings provide evidence that the overexpression of exo-miR-210-5p inhibits chondrocytes energy metabolism by suppressing basal and max aerobic respiration. MiR-210-5p also contributes to OA related genes alteration in chondrocytes by upregulating hypertrophic genes while inhibiting chondrogenic genes expression, which might further result in cartilage degradation. The ability of targeting hundreds or even thousands of genes is a common characteristic of a microRNA. As the first small-interfering RNA, Patisiran, was recently granted FDA approval in 2018, studies focused on the preclinical and clinical applications by microRNA were expanded [45]. There is also clinical utility reported of using miRNA repressors to silence the transcript function in peripheral tissues [46]. Of note, several possible approaches for microRNA could be summarized as (1) biomarkers for pathogenic conditions; (2) modulators of drug resistance; (3) small molecule drugs for medical intervention in almost all human health conditions [39,47,48]. As subchondral bone changed earlier than cartilage during OA progress, the abnormal upregulation of miR-210-5p in SBOs derived exosomes could be used as a marker/predictor for OA. Inhibitors suppressing the expression of miR-210-5p in SBOs could be a novel direction for OA therapy.

In conclusion, this study showed that exosomal miRNAs play a central role in communication between cartilage and subchondral bone.

## Data Availability

The data presented in this study are available on request from the corresponding author. The data are not publicly available due to restrictions eg privacy or ethical.

## Supplementary Materials

The following are available online at https://www.mdpi.com/2073-4409/10/2/251/s1, Figure S1: MicroRNA targeted pathway analysis., Table S1: MicroRNA primers sequence.
