# Supplementary material for: Osteoarthritic Subchondral Bone Release Exosomes That Promote Cartilage Degeneration
Source: Cells. 2021 Jan 28;10(2):251. doi: 10.3390/cells10020251 (PMC7911822; doi:10.3390/cells10020251)
Supplement: Supplementary file 1 [file cells-10-00251-s001.pdf]

Supplementary Materials and Figures:

**miRNA**<br><br>**Copyright: © 2021 by the authors. Licensee MDPI, Basel, Switzerland. This article is an open access article distributed under the terms and conditions of the Creative Commons Attribution (CC BY) license (http://creativecommons.org/licenses/by/4.0/).**	**Sequence**
hsa-miR-92a-3p	5'-UAUUGCACUUGUCCCGGCCUGU-3'
hsa-miR-5087	5'-GGGUUUGUAGCUUUGCUGGCAUG-3'
hsa-miR-210-5p	5'-AGCCCCUGCCCACCGCACACUG-3'
hsa-miR-670	5'-GUCCCUGAGUGUAUGUGGUG-3'
hsa-miR-4266	5'-CUAGGAGGCCUUGGCC-3'
hsa-miR-135a-3p	5'-UAUAGGGAUUGGAGCCGUGGCG-3'

Table Supplementary Table 1. miRNA primers sequence

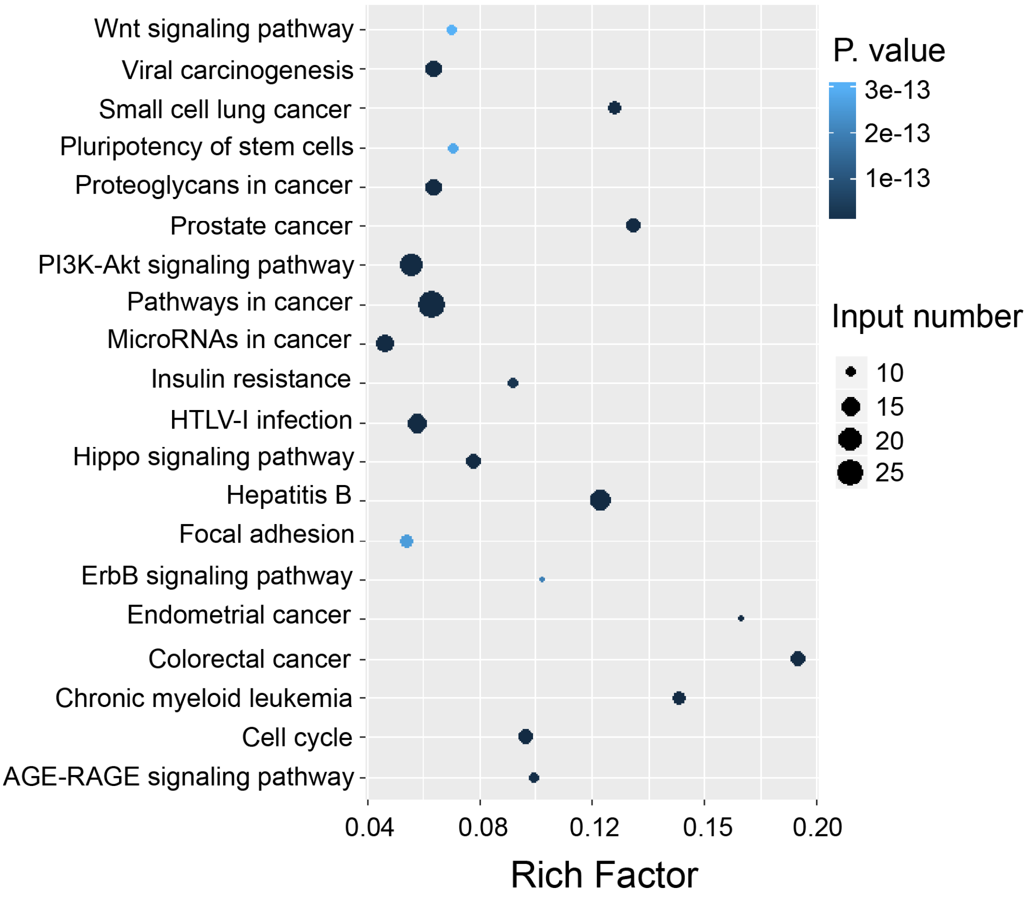

Figure Supplementary Figure 1. MicroRNA targeted pathway analysis. Kyoto Encyclopedia of Genes and Genomes database pathway analysis showed that these microRNAs were corresponded to cell cycle, PI3K-Akt, Hippo and AGE-RAGE signaling pathway.
